# Supplementary material for: Vascular Microbleeds Without Brain Atrophy: A Microvascular Signature of Mid-Stage 5xFAD Pathology
Source: bioRxiv. 2025 Dec 11:2025.12.08.692809. Preprint. [Version 1] doi: 10.64898/2025.12.08.692809 (PMC12713755; doi:10.64898/2025.12.08.692809)
Supplement: Supplement 1 [file media-1.pdf]

Vascular Microbleeds Without Brain Atrophy: A Microvascular Signature of Mid-Stage 5xFAD Pathology

Xiuli Yang, Yuguo Li, Adnan Bibic, Jiekang Wang, Mei Wan, Wenzhen Duan,
Hanzhang Lu, Zhiliang Wei

Supplemental material

1. Immunohistochemistry image of Prussian blue staining in an intracerebral hemorrhagic stroke model

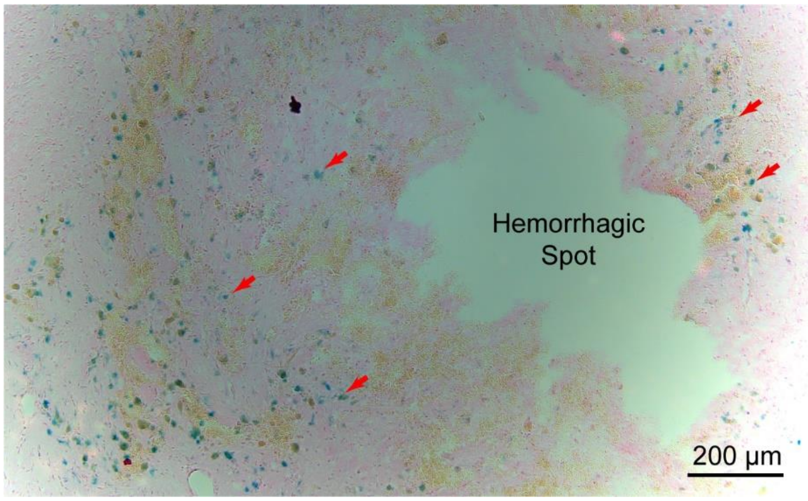

Figure S1. Prussian blue staining of a brain section from a mouse with intracerebral hemorrhagic stroke. The stroke model was generated by intra-striatal injection of collagenase. Red arrows indicate exemplary regions showing positive Prussian blue staining, corresponding to iron deposition.

2. DWI quantification in the CADASIL model

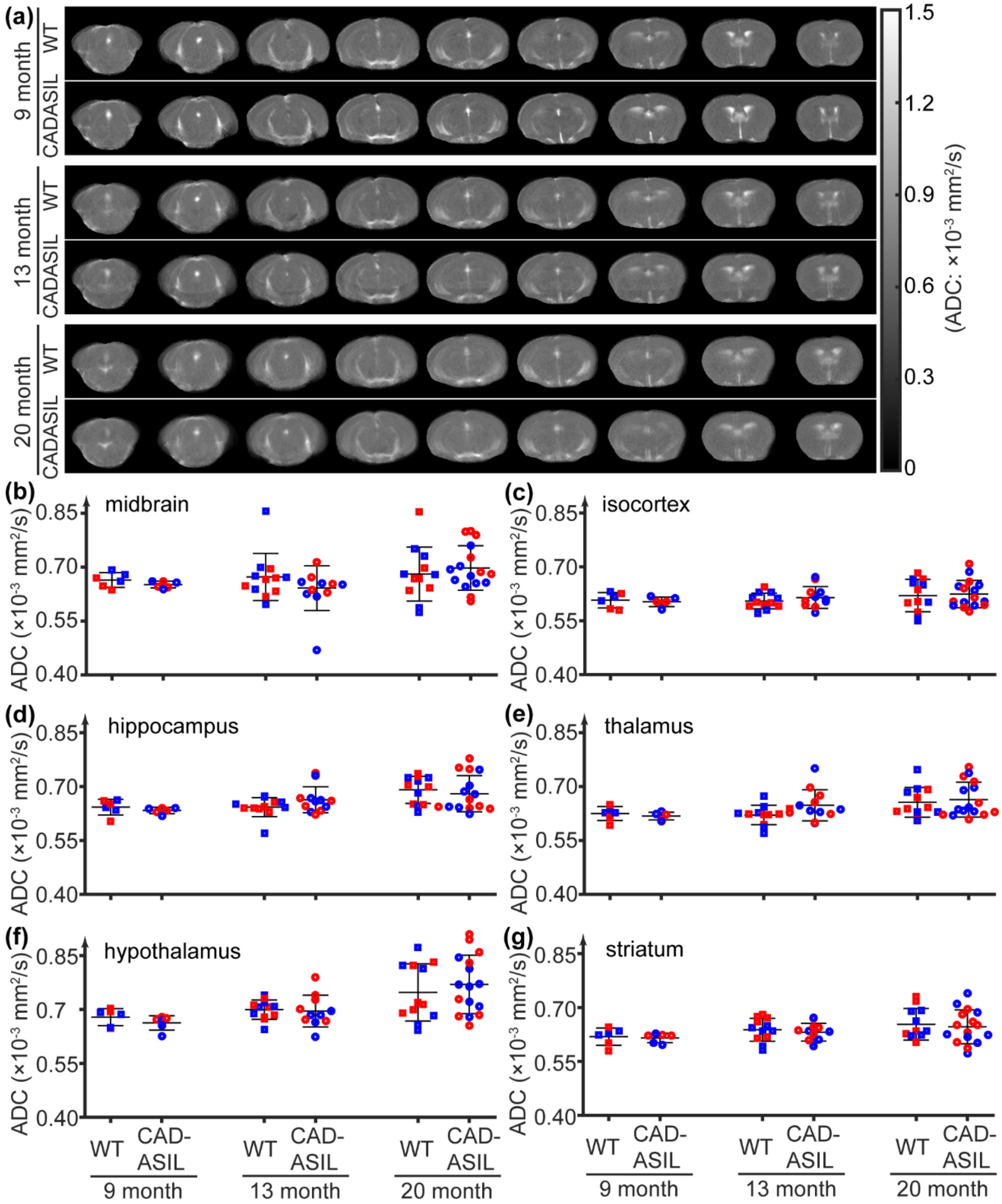

Figure S2. Averaged maps and regional quantification of ADC in the CADASIL model at 9, 13, and 20 months of age. (a) Averaged ADC maps. (b-g) Regional ADC across the tested time points for midbrain, isocortex, hippocampus, thalamus, hypothalamus, and striatum, respectively. Red and blue dots represent female and male mice, respectively.

3. Statistical analyses of regional ADC values in the CADASIL model

Table S1 Detailed information of linear mixed-effects models

Region	ADC ~ genotype + age + sex + (1|mouse)									ADC ~ genotype × age + sex + (1|mouse)		
	genotype			age			sex			genotype × age		
	β (×10^{-5} mm^2/s)	95% CI (×10^{-5})	*p*	β (×10^{-6} mm^2/s/ month)	95% CI (×10^{-6})	*p*	β (×10^{-5} mm^2/s)	95% CI (×10^{-5})	*p*	β (×10^{-6} mm^2/s/ month)	95% CI (×10^{-6})	*p*
midbrain	-0.65	[-3.59, 2.28]	0.657	**3.58**	**[0.24, 6.94]**	**0.036**	1.43	[-1.49, 4.35]	0.332	4.05	[-2.58, 10.70]	0.227
isocortex	0.49	[-1.18, 2.15]	0.562	1.49	[-0.21, 3.19]	0.085	-0.21	[-1.87, 1.45]	0.799	0.16	[-3.26, 3.57]	0.927
hippocampus	0.23	[-1.69, 2.16]	0.808	**3.85**	**[2.02, 5.69]**	**< 0.001**	0.44	[-1.48, 2.35]	0.650	-3.12	[-6.51, 0.26]	0.070
thalamus	1.35	[-0.70, 3.41]	0.193	**2.80**	**[1.09, 4.51]**	**0.002**	-0.61	[-2.65, 1.44]	0.552	-1.85	[-5.07, 1.36]	0.254
hypothalamus	-1.83	[-7.23, 3.58]	0.501	**7.89**	**[2.93, 12.84]**	**0.002**	3.97	[-1.42, 9.35]	0.146	5.95	[-3.68, 15.6]	0.221
striatum	-0.62	[-2.43, 1.19]	0.493	**2.38**	**[0.51, 4.24]**	**0.014**	0.58	[-1.22, 2.39]	0.521	-0.27	[-4.02, 3.48]	0.888
